# Activation Capacity of the Intrinsic Musculature of the Foot in Handball Athletes with Chronic Ankle Instability

**DOI:** 10.3390/biomedicines11082115

**Published:** 2023-07-27

**Authors:** Daniel García-García, Rocío Llamas-Ramos, César Calvo-Lobo, David Rodríguez-Sanz, Marta San Antolín-Gil, Juan Luis Cabanillas-García, Mari Cruz Sánchez-Gómez, Inés Llamas-Ramos

**Affiliations:** 1 IMSKE Hospital, 46024 Valencia, Spain; 2 Faculty of Nursing and Physiotherapy, Universidad de Salamanca, 37007 Salamanca, Spain; 3 Facultad de Enfermería, Fisioterapia y Podología, Universidad Complutense de Madrid, 28040 Madrid, Spain; 4 Departamento de Psicología, Universidad de Valladolid, 47011 Valladolid, Spain; 5 Departamento de Didáctica, Organización y Métodos de Investigación, Universidad de Salamanca, 37008 Salamanca, Spain; 6 University Hospital of Salamanca, 37007 Salamanca, Spain; 7 Institute of Biomedical Research of Salamanca (IBSAL), 37007 Salamanca, Spain

**Keywords:** ankle instability, ultrasound, dynamometry, handball, diagnosis, prevention

## Abstract

Chronic ankle instability (CAI) is a common pathology in handball. The role of the intrinsic musculature of the foot in these players in relation to proprioception and stability has not been stablished. The objective of this study was to compare the ultrasound morphology of the heel fat pad of the foot in professional handball players to CAI in healthy players and establish relationships between CAI and physical and psychological variables. The study has been a descriptive observational case–control study in which 20 professional handball male players over 18 years of age were divided into 8 cases (CAI) and 12 controls (healthy). An ultrasound evaluation, the thickness and/or cross-sectional area at rest and the contraction measurement of the Abductor Digiti Minimi, Abductor Hallucis, Flexor Digitorum Brevis and Quadratus Plantae muscles were analyzed. Moreover, the compressibility index of the heel fat pad and the activation of the abdominal wall musculature (ultrasound), the flexion strength of the hallux and lesser toes (dynamometry), the foot functionality (Bristol Foot Score (BFS) questionnaire) and the psychological variables (self-reported questionnaires) were measured. There were no significant differences between the activation capacity values of the foot muscles of healthy and CAI athletes. Significant differences were found between groups regarding the BFS score (*p* = 0.007), d = 1.404), and significant correlations were also found between hallux flexion strength and lesser toes flexion strength in the total sample. Although there were no differences between the two groups, the identification of the activation pattern of these muscles in handball athletes is essential to the improvement of performance and preventing injuries such as CAI.

## 1. Introduction

Handball is a contact sport in which several actions take place that can be related to an injury risk for players, such as jumps, landings, imbalances, feints and throws; these actions make handball one of the sports with the highest injury incidence [1]. Between 2008 and 2011, an article related to 35 different sports, like soccer, handball, ice hockey and floorball, reported a handball injury incidence of 63.4%. Moreover, the highest incidences of time off work caused by sports were found for the following sports: motorcycling, handball, skating and ice hockey [1]. Another study by Engebretsen et al. [2] evaluated all injuries and illnesses recorded during the London 2012 Olympic Games. Among men’s sports, taekwondo (51.5%), BMX (Bicycle Motorcross) (31.3%), soccer (27%) and handball (17.4%) athletes experienced the highest prevalence of injuries. As for severe injuries, 3.4% of handball players suffered such, and this rate was only lower than the incidences recorded in taekwondo (7.8%), BMX (6.3%), basketball (4.2%), weightlifting (4%), triathlon (3.6%) and judo (3.5%) athletes.

Several studies have recorded the injuries produced during different tournaments or throughout a professional sports season, and it has been observed that 17.4–19.4% of acute or traumatic handball-related injuries affect the ankle [3,4,5,6]. The ankle is the anatomical region with the highest incidence of injury in handball, according to these studies, ahead of the knee (11.4–26%) and thigh (9.7–15.9%). Specifically, the sprain of the external lateral complex (EEL) of the ankle is the most frequently recorded injury (26 out of 28 ankle injuries were ELL-related injuries (external lateral ligament) sprains) [7], and these ELL injuries represented up to 80% of all injuries [8]. In addition, two other studies have reported that 7.3% [9] and 8% [10], respectively, of all injuries were ankle ELL sprains, and the rate of recurrence ranges from 40 [7] to 50% in Seil’s study [11], causing high functional instability and discomfort that remains for up to 6 months after the injury in up to 40% of cases [12]. 

Ultrasonography is an indirect method used to assess muscle mass that has excellent intra-observer reliability [13,14]. The mass estimation is performed by measuring the thickness and cross-sectional area of the muscle belly. Specifically, at the level of the foot intrinsic musculature, alterations in its morphology have been found in patients with Achilles tendinopathy [15], post-stroke conditions [16] and flat feet [17], finding, in some cases, a significant correlation between these alterations and the strength loss of the flexor musculature of the toes [18]; despite representing a different population, this trend could also be present in handball players.

The weakness of the intrinsic musculature of the foot is related to various pathologies, although no articles have been found that clearly establish the relationship between this weakness and ligament injuries in the ankle. The economic impact that ankle sprains have on health systems or sporting performance, as well as their prevalence, make necessary our research into the prevention and treatment of ankle sprains and their sequelae, especially chronic instability. The main objective of this study was to compare the ultrasound morphology of different muscles in the foot sole in professional handball players to chronic ankle instability and healthy players, as well as to establish relationships between chronic ankle instability and several physical and psychological variables based on the hypothesis that the heel fat pad thickness has a relationship with the activation capacity of the intrinsic musculature of the foot.

## 2. Materials and Methods

### 2.1. Design

An observational descriptive case–control study was carried out that followed the Strengthening the Reporting of Observational Studies in Epidemiology (STROBE) criteria [19] for comparison between various physical and psychological factors in athletes with chronic ankle instability and healthy athletes.

### 2.2. Participants

The sample was composed of professional handball male players aged between 18 and 40 years old. All athletes were senior players, having more than 10 years of experience in this sport. They were divided into an experimental group (cases: professional handball players with chronic ankle instability diagnosed by a doctor) and a control group (athletes without this pathology).

The inclusion criteria were as follows: being older than 18 years old; being a professional handball player with chronic ankle instability diagnosis, according to the criteria of the International Ankle Consortium [20], for the experimental group (cases); and, in the control group, being a healthy handball player without ankle alterations who possessed the same level of sporting experience as members of the experimental group.

The exclusion criteria established were having previous surgery or fractures in any of the lower extremities; having an acute musculoskeletal injury in the ankle in the last 3 months, with resulting physical activity cessation (at least 1 day); and the presence of neurological or vestibular diseases.

The study was conducted between November 2020 and February 2021. The recruitment was carried out at the European Musculoskeletal Institute of Valencia/Kineos Physiotherapy Center/Sports Club. Data collected were related to age, sex, body mass index (BMI) weight, height, time of sports practice, dominant lower extremity, playing position, use of insoles, use of ankle bandage or orthosis and foot posture.

Approval was obtained from the Ethics Committee of the Clinical Hospital San Carlos (Madrid) (20/478-E). The basic principles of the Declaration of Helsinki have been respected. The information sheet and informed consent form signed by subjects in order to be part of the research were explained to the participants prior to the experiment. Likewise, they could sign a revocation form at any time. The participants’ data were secured by the principal investigator and/or research team. The patients were coded using numbers ranging from 01 to 20 to ensure confidentiality and data anonymity.

### 2.3. Outcome Measures

#### 2.3.1. Ultrasound Morphology

**Figure 1:**
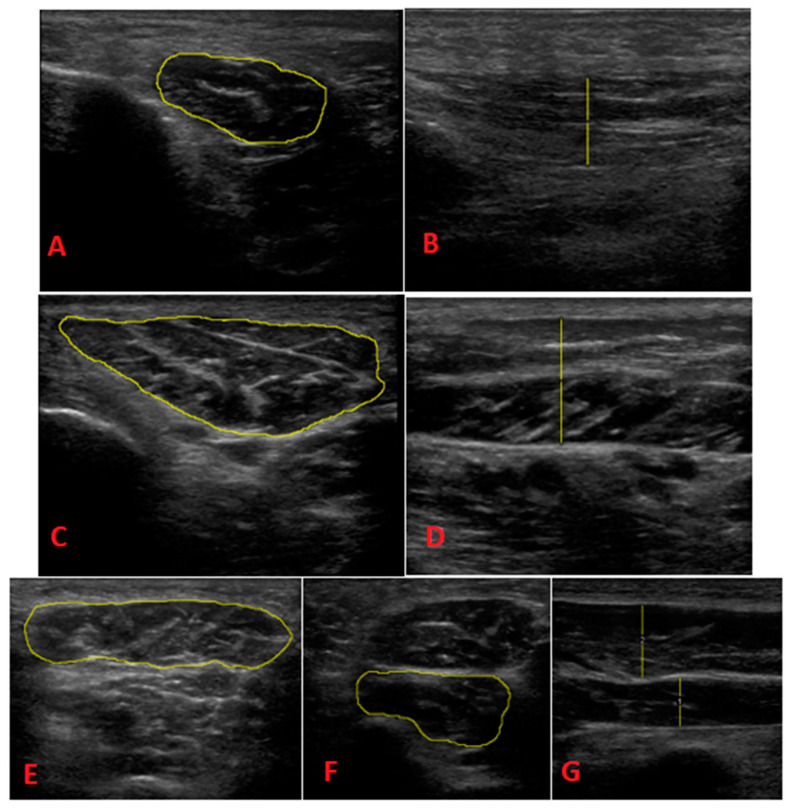
(**A**) (AbDM CSA); (**B**) (AbDM grosor); (**C**) (AbH CSA); (**D**) (AbH thickness); (**E**) (FDB CSA); (**F**) (QP CSA); (**G**) (QP and FDB thickness). AbDM (abductor digiti minimi muscle); AbH (abductor hallucis muscle); FDB (flexor digitorum brevis muscle); QP (quadratus plantae muscle). CSA (cross-sectional area).

The experiment’s main outcome was the determination of the ultrasound morphology (GE Healthcare, Wisconsin, USA) of the flexor hallucis brevis (FHB), abductor hallucis (AbH), abductor digiti minimi (AbDM), quadratus plantae (QP) and flexor digitorum brevis (FDB) muscles, as we assessed the thickness and cross-sectional area of their muscle bellies both at rest and in contraction (Figure 1). A General Electric^®^ Logic-E ultrasound scanner with a 12L-RS (8–13 MHz) linear probe was used to perform ultrasound evaluation. The necessary images were taken in B mode. Three images of each structure were taken, and we lifted the probe between taking each image. For the analysis, the average value of the 3 measurements obtained was selected. It was necessary to avoid putting pressure on the probe.

In the case individuals, the foot on the side of the affected ankle was assessed, while in the controls, the foot assessed was randomly selected.

Once all images were acquired, measurements were performed using Image J software version 2.0 (National Institutes of Health, Bethesda, MD, USA). Perimuscular fascial tissue was not included in the thickness and CSA measurements. In addition to ultrasound measurements, the contraction ratio was calculated by dividing the contraction measurement result by the resting measurement result.

To take the ultrasound images, the protocol defined by Mickle et al. [14] was followed:

- FHB: Each patient was assessed in the prone decubitus position. We placed the probe longitudinally into the first MT (slightly oblique posterolateral direction). We then performed a proximal sweep to locate the thickest portion of the muscle belly, which was distal to the base of the first MT. The image was taken to measure the thickness.
- AbH: Each patient was assessed in the supine position with slight external rotation of the hip and slight knee flexion. We placed the probe into the medial tuberosity of the calcaneus toward the scaphoid tubercle. Normally, the area of greatest thickness is located 1–2 cm proximal to the scaphoid tubercle. A longitudinal image was taken to determine thickness and a transverse image was taken to determine CSA.
- AbDM: Each patient was assessed in the prone position. We located the insertion of the muscle in the lateral calcaneal tubercle and oriented the probe toward the tuberosity of the 5th MT. Normally, the area of greatest thickness is located near the calcaneocuboid joint, which was found before the tendon appeared. Longitudinal acquisition was performed to measure thickness and transverse acquisition was performed to determine CSA.
- QP: Each patient was assessed in the prone position. The quadratus plantaris was located deep in the flexor digitorum brevis. We then located the talus-calcaneus-scaphoid joint and used the longitudinal probe to align in the direction of the muscle fibers, looking for the area of greatest thickness in the muscle belly, which is usually found proximal to the spring ligament. We then measured the thickness in the longitudinal section and measured CSA in the transverse section.
- FDB: Each patient was assessed in prone position. We drew a line between the medial calcaneal tubercle and the third toe. We placed the probe in a longitudinal position relative to this line, which extended from the insertion in the calcaneus, and we performed distal sweeping until locating the area of greatest thickness in the muscle belly, before dividing it into 4 fascicles. The image was longitudinally taken to determine the thickness in the longitudinal section and transversely taken to determine the thickness in the ASC.

The remaining variables were measured as follows:

- Heel fat pad morphology: The longitudinal section was measured at the calcaneus level and in the area of greatest thickness of the heel fat pad [21]. Measurements were first taken without compression and then taken with compression. The distance between the skin and the plantar fascia (heel fat) was measured. The compressibility index was calculated by dividing the thickness in compression by the thickness without compression. The measurements of the heel fat thickness were also determined using Image J 2.0 software (National Institutes of Health, Bethesda, MD, USA), as was the intrinsic musculature determined via the foot assessment.
- Abdominal wall thickness: A bilateral ultrasound assessment was performed at rest (at the end of a relaxed exhalation) and at the contraction (homolateral hip flexion with the knee extended) of the abdominal wall thickness muscles and the inter-rectus distance. Three points were recorded: the anterolateral abdominal wall (thickness of the EO, IO and TrA), the anterior abdominal wall (rectus abdominis thickness) and the anterior abdominal wall 2 (inter-straight distance).
  - Anterolateral abdominal wall: Each patient was assessed in the supine position with a slight flexion of the hips and knees, with a cushion placed in the popliteal fossa. We transversally placed the probe at the level of the mid-axillary line at the midpoint between the lower edge of the costal grid and the iliac crest. In this way, we measured the thicknesses of the EO, IO and TrA.
  - Anterior abdominal wall: Each subject was assessed in the supine position with slight flexion of the hips and knees, with a cushion placed in the popliteal fossa. We transversely place the probe at the level of the umbilicus and laterally to the midline. In this image, we can see the transverse section of the rectus abdominis, of which we measured the thickness.
  - Anterior abdominal wall 2: Each subject was assessed in the supine position with hips and knees flexed, with a cushion placed in the popliteal fossa. We transversely placed the probe on the midline, which was just proximal to the umbilicus. In this image, we measured the inter-rectal distance. This distance was calculated from one muscle belly to the other, i.e., the connective tissue was included in the distance (the limits of the inter-rectus distance would be the muscle bellies themselves).

#### 2.3.2. Foot Functionality: Bristol Foot Score Questionnaire

This questionnaire consisted of 15 questions that assessed pain issues (7), mobility and footwear (3), general foot health (4) and general health status (1). Different scores were given depending on the question (1–9). The higher the score, the greater the impact of the foot on the subject’s functionality [22].

#### 2.3.3. Toe Flexor Strength

Dynamometry was used to assess the strength of flexion of the MTF joints while the IFs were extended [13,23] using a Lafayette Hand-Held dynamometer (Lafayette Instrumemt Company, modelo 01165, Lafayette, IN, USA). The procedure for assessing the strength of the toe flexors involved placing the dynamometer under the IF joint of the hallux first and the other toes second. We asked each subject to exert as much force as possible to bend the toes against the dynamometer. Indeed, the movement based on which we assessed the force was the bending of the MTF joints, thus keeping the IF joints extended [13,23].

#### 2.3.4. Psychological Outcome Measures

Psychological outcome measures were measured using the State-Trait Anxiety Inventory, the Beck Depression Inventory, the Tampa Scale for Kinesiofobia and the Spanish version of Eysinck.

The State-Trait Anxiety Inventory (STAI) measures two dimensions of anxiety: trait-anxiety, which determines the permanent anxiety level, and state-anxiety, which measures how the person feels at that moment. Each dimension has 20 items on a Likert-type scale of 0–3 (not at all to very much). The higher the score, the greater the presence of anxiogenic symptoms [24]. The Beck Depression Inventory (BDI-II) presents 21 Likert-type items related to sadness, pessimism, feelings of failure, loss of pleasure, feelings of guilt, feelings of punishment, etc. Each item has a four-point scale (0, 1, 2, 3), except items 16 and 18, which each have 7 categories. The total score is categorized into minimal, mild, moderate or severe depression, with a higher score linked to greater severity [25,26]. A Spanish study’s adaptation of the Tampa Scale for Kinesiophobia (TKS-11) [27] assessed fear and avoidance beliefs regarding pain. It consists of 11 Likert-type items from 1 to 4 (strongly disagree to strongly agree). It is divided into two subscales: activity avoidance and harm. A higher score means greater fear of pain and avoidance of movement [28,29]. An abbreviated and adapted Spanish version of the Eysenck (EPQ-RA) [30] assesses neuroticism, psychoticism, extraversion and dissimulation/conformity based on 24 items. It evaluates these 4 dimensions using 6 items per scale and two YES/NO response alternatives. The greater the number of affirmative answers, the greater the presence of each dimension in the subject’s personality [31].

### 2.4. Procedure

The study was carried out at the facilities of the European Musculoskeletal Institute of Valencia/Kineos Physiotherapy Center/Sports Club by a musculoskeletal ultrasound specialist with more than 5 years of experience, who performed ultrasound imaging (the intrinsic musculature of the foot, the heel fat and the abdominal wall musculature), strength assessments (toe flexors dynamometry) and Foot Posture Index-based tests. The evaluator did not know before performing the assessment whether the subject was considered to be a case or control participant. After the measurements were taken, a personal interview was conducted with each player, in which the demographic and personal variables were obtained and the subject was defined as a case or control participant (30–45 min in total). Each individual was also given a notebook with the self-reported tests (Bristol Foot Score, Cumberland Ankle Instability Tool, STAI, BD I-II, TSK-11 and EPQ-RA), which they completed and handed to the evaluator; this process had an average duration of 15–20 min. Finally, another researcher performed the evaluation of ultrasound images, determining the thickness and cross-sectional area of the assessed musculature and the thickness of the heel fat pad. A third investigator interpreted the results of the psychological assessment tests. 

### 2.5. Statistical Analysis

All data were compiled into a database, and SPSS software, version 22.0 for Windows (SPSS Inc., Chicago, IL, USA) was used. An α error of 0.05 (95% confidence interval [CI]), which had a desired power of 80% (β error of 0.2), was established. To determine the type of statistical test, the Shapiro Wilks statistical test was performed (because the number of observations was less than 50) to check the normality of each of the data series. All of them obtained a *p* value < 0.05; thus, they met the principles of normality, meaning that the non-parametric Mann Whitney U test was used. This test described the median and interquartile range (for non-parametric data) that were selected to compare the data belonging to each group. For categorical qualitative variables, Fisher’s exact test for dichotomous variables and the Chi-square test (χ2) for polytomous variables, which were described using frequencies and percentages, were used to compare both groups.

To calculate the effect size, Cohen’s D statistic and Matthew’s Correlation Coefficient for those contrasts of *p* > 0.05 were obtained. The calculations were performed using G*Power software, version 3.1.9.7 (Dusseldorf University, Dusseldorf, Germany) [32]. For the interpretation of the effect size, the classification determined in [33] was used for the contrast between independent groups (d = 0.200 = small; d = 0.500 = medium; d = 0.800 = large) and the contrast between correlations (φ = 0.100 = small; φ = 0.300 = medium; φ = 0.500 = large). 

## 3. Results

A total of 20 participants (*n* = 20), who were professional male handball players with either chronic ankle instability (case group, *n* = 8) or good ankle health (control group, *n* = 12), were evaluated. They had an average age 26.85 and an average of 16.1 of years of practice.

### Descriptive Data Analysis

Table 1 shows the descriptive results for each of the variables analyzed. The cases group obtained significantly higher mean scores in BRISTOL (31.875/21.083), F. HALLUX (169.565/138.849) and F. FINGERS (162.92/128.162). On the other hand, the control group obtained a significantly higher mean score in F. SENSE (1.293/0.643). For the remaining variables of foot ultrasound morphology, astragal fat morphology and abdominal wall thickness, no major differences between mean values were observed. Secondly, the results of the descriptive analysis of the psychological test variables, which we used to highlight higher mean scores in the cases group in psychoticism (1.375/1.083) and extraversion (4.250/3.750), are shown. Regarding the control group, we obtained higher mean scores for neuroticism (1.500/1.125), sincerity (4.333/3.250), avoidance (13.917/12.125), state-anxiety (26.750–73.083/26–71.375) and trait-anxiety (28–78.417/23.875–68.125). 

Differences between the case/control groups were analyzed in relation to the ultrasound morphology and psychological test variables. It can be seen in Table 1 that statistically significant differences were only found in BRISTOL (*p* = 0.007), which had a large effect size (d = 1.404) and applied an α = 0.05 power (1-β probable error = 0.80); these results could be generalizable for *n* = 20, case = 10 and control = 10.

In addition, no statistically significant differences were found for the remaining ultrasound morphology variables of the foot (*p* > 0.05). 

Regarding the differences between the case and control groups related to the psychological test variables, no statistically significant differences were found (*p* > 0.05).

Table 2 shows the significant correlations found between the foot morphology variables. Of note is the high positive correlations between F-HALLUX-F-ToES (r = 0.916; *p* = 0.000; φ = 0.957), FPI-TrA D (r = 0.600; *p* = 0.005; φ = 0.774) and OE D-OE I (r = 0.589; *p* = 0.006; φ = 0.767). Among these negative and significant correlations, those found between GROSOR AbH-OI I (r = −0.540; *p* = 0.014; φ = 0.734) and CSA FDB-RA I (r = −0.525; *p* = 0.017; φ = 0.724) are noteworthy. Regarding the remaining the correlations analyzed in relation to the foot morphology variables, no statistically significant correlations were found. All correlations have a large effect size.

Regarding the analyses used to determine correlations between the psychological test variables, we found positive and significant correlation between EPQ-RA Neuroticism-BD I-II (r = 0.647; *p* = 0.002; φ = 0.804) and STAI A/E-STAI A/R (r = 0.697; *p* = 0.001; φ = 0.834). Both correlations have a large effect size.

## 4. Discussion

The main objective of the study was to determine the differences in IPM activation capacity between subjects with IAC and healthy subjects. To the best of the authors’ knowledge, this experiment was the first study performed with this objective that used a sample of professional athletes (handball players).

Regarding the activation capacity of the IPM, no significant differences were observed between cases and controls. Indeed, Fraser et al. [34] did not find correlations between the sizes of the FHB, FDB, QP and AbH muscles and the probability of experiencing prior ankle sprains or CAI, and Feger [35] found that CAI subjects had smaller muscle volumes in the oblique heads of the hallux adductor and FHB than healthy subjects. Moreover, no significant differences between groups were found in the AbH, AbDM, FDB or QP muscles. This study assessed muscle volume with MRI, rather than ultrasound, and used a small sample of 5 cases and 5 controls. Furthermore, unlike our study, only muscle volume at rest was assessed, while muscle activation capacity was not assessed.

The role of IPM may be relevant in a pathology such as CAI. Lee et al. [36] performed a study that included a sample of 30 subjects with CAI, (control and case group) and these participants underwent an IPM-strengthening program for 6 weeks. The activation ratios of the AbH, FDB, FHB and QP muscles were measured via ultrasound before and after the program. The case group increased the activation ratio of the assessed muscles, which correlated with a significant improvement in dynamic stability. Nevertheless, the pre-training activation ratios of AbH, FDB, FHB and QP were different in this study to our investigation.

Foot function assessed via self-reported BFS questionnaire, which showed that the mean score in the case participants was 31.8 ± 8.7 points, while in the controls, it was 21.0 ± 6.5. This finding is consistent with those found in previous studies that compared subjects with IAB and healthy subjects, like Wikstrom and Song [37], who used the FAAM questionnaire (FAAM-ADL: controls 99.94 ± 0.24 and cases 86.98 ± 10.48; FAAM-S: 100.00 ± 0.00 and 76.33 ± 15.66), and Kazemi et al. [38], who used the FAOS questionnaire, which demonstrated a decrease in foot function in subjects with IAC compared to healthy subjects. On the other hand, in this study, it has been found that subjects with CAI show higher flexion strength levels than controls for the hallux (169.5 ± 36.0 N vs. 138.8 ± 55.2 N) and the lesser toes (162.9 ± 38.1 N vs. 128.1 ± 38.7 N). This finding contrasts with Frasser’s study [39], which also assessed finger flexion strength with dynamometry, that found that the strength values of controls were higher than those of CAI subjects. The increased strength of the toe flexors in subjects with CAI found in the present study could be explained based on a compensatory mechanism that compensated for the lack of ankle stability through IPM activation. This process was be related to proprioception, rather than purely IPM motor function, as highlighted in the study by Lee et al. [40]. Comparing subjects with CAI who performed an 8-week program of Short Foot Exercise to another group that performed proprioceptive sensory exercise for the same period, it was found that the group that performed the Short Foot Exercise obtained greater joint position sense, vibration sensitivity threshold, dynamic stability and self-perceived improvements in ankle stability.

According to Hertel’s updated CAI model [41], some of the key components in CAI are personal and environmental factors; thus, it was decided to include some psychological variables in the present study. Higher values of psychoticism and extraversion in cases and higher values of neuroticism, sincerity, avoidance of movement and anxiety in controls were found. Other studies comparing CAI and healthy subjects showed significant differences regarding fear of injury (FABQ-PA questionnaire) (4) or kinesiophobia (5), as the CAI group had TSK-11 values of 22.6 ± 6.0, compared to 17.4 ± 5.8 in the control group.

On the other hand, no significant differences were found regarding foot shape as measured via FPI. This finding agrees with other studies that compared CAI subjects or sprains to healthy subjects [39,42]. In contrast, Mei Dan et al. [43] conducted a retrospective analysis that identified correlation between foot shape and history of ankle sprains. However, CAI subject’s inclusion criteria are not defined, being based solely on self-reported history of sprains. Foot posture measurement was not performed with FPI but with the Chippaux–Smirac Index (correlation between lower longitudinal arch and a clear history of sprains was defined [44].

Total sample results found that hallux flexion strength and lesser toes flexion strength were directly correlated, as found by Abe et al. [45]. Furthermore, they also found no correlation between toe flexion strength and resting CSA of AbH and FDB muscles in healthy men. In contrast, in the present study, we proved that a correlation existed between the flexion strength of the lesser fingers and the activation capacity of the QP (by assessing its CSA).

Some correlations were found between abdominal wall activation and other foot-related variables, such as IPF (direct correlation with activation of the transverse abdominis and OI), talar fat (direct correlation with activation of the transverse abdominis) and muscle activation in the CSA measurement of FDB and QP and AbH thickness (negative correlation with abdominal muscle activation). Although these correlations are statistically significant, considering the sample sizes, they should be analyzed with caution. Some authors established relationships between the abdominal wall and MIP or dynamic stability. Sanchez et al. [46] (pronated foot sample) found improvement in foot posture (tendency to neutralize the stride, which rose from an FPI score of 8.1 ± 1.7 to a score of 6.4 ± 2.1 points) thanks to the implementation of a strengthening program applied to the foot and the lower limb musculature and core. Ozmen and Aydegmus [47] applied a core strengthening program to healthy athletes, finding, at the end of the program, an improvement in dynamic stability. Finally, Jiang et al. [48] report improvements in ankle strength, jumping tests and dynamic stability following a Pilates training program in which CAI subjects participated. Therefore, it is recommended that we continue evaluating the possible relationships between the abdominal wall musculature and IPM and CAI.

The main limitation of the study is its small sample size. Furthermore, only male players were included in the sample. Moreover, given the clinical condition established in the present study (CAI), it is impossible to perform randomization or blind the evaluator. 

It is advisable, in future studies, to increase the sample size and include female athletes. The blindness of the evaluator to the initial diagnosis of each patient must be established, and we need to investigate this potential relationship in other sports.

## 5. Conclusions

There are no significant differences in the activation capacities of the AbDM, AbH, FDB and QP muscles between healthy handball players and players with chronic ankle instability. However, a correct and initial diagnosis could help physiotherapists and physical trainers to establish better treatments and training regimens. The identification of the activation pattern of these muscles in handball athletes is essential to improving player performance and preventing injuries such as CAI.

## Figures and Tables

**Table 1:** Descriptive and inferential results of the ultrasound foot morphology variables and psychological tests.

Variables	Group	Mean	S_x_	Maximum	Minimum	Significance	d
BFS	Control	21.083	6.501	35	15	0.007 *	**1.404**
	Case	31.875	8.709	48	20		
FPI	Control	3.083	3.704	7	−3	1.000	0.020
	Case	3.000	4.309	8	−6		
BMI	Control	24.980	2.418	28	21	1.000	0.268
	Case	25.763	3.332	33	23		
HALLUX F.	Control	138.849	55.277	272.13	72.6	0.069	0.658
	Case	169.565	36.019	216.73	128.73		
TOES F.	Control	128.162	38.779	197.4	76.2	0.115	0.903
	Case	162.920	38.183	219.53	115		
SENSE F.	Control	1.293	3.496	9.29	−2.37	0.970	0.201
	Case	0.643	2.927	6.59	−3.53		
THICKNESS AbH	Control	1.035	0.018	1.073	1.012	0.734	0.419
	Case	1.048	0.04	1.108	1.003		
CSA AbH	Control	1.055	0.028	1.093	1.016	0.910	0.148
	Case	1.059	0.026	1.107	1.031		
THICKNESS AbDM	Control	1.072	0.050	1.178	0.998	0.851	0.209
	Case	1.086	0.080	1.261	1.006		
CSA AbDM	Control	1.137	0.122	1.384	0.996	1.000	0.048
	Case	1.131	0.126	1.408	1.015		
THICKNESS QP	Control	1.092	0.081	1.262	1.009	0.157	0.729
	Case	1.024	0.104	1.141	0.781		
CSA QP	Control	1.149	0.088	1.304	1.017	0.473	0.377
	Case	1.116	0.087	1.280	0.979		
THICKNESS FDB	Control	1.097	0.047	1.188	1.029	0.384	0.080
	Case	1.091	0.095	1.304	0.990		
CSA FDB	Control	1.053	0.026	1.102	1.015	0.910	0.299
	Case	1.071	0.081	1.229	0.990		
HEEL FAT	Control	0.699	0.067	0.797	0.596	0.427	0.422
	Case	0.674	0.050	0.758	0.606		
IRD	Control	0.112	0.213	0.718	−0.136	0.792	0
	Case	0.112	0.124	0.377	−0.004		
RA R	Control	1.054	0.053	1.177	0.995	0.157	0.480
	Case	1.079	0.051	1.164	1.019		
RA L	Control	1.063	0.040	1.145	1.016	0.734	0.118
	Case	1.067	0.026	1.107	1.018		
TrA R	Control	1.112	0.093	1.259	0.984	0.734	0.242
	Case	1.137	0.112	1.374	1.035		
TrA L	Control	1.125	0.126	1.404	1.008	0.851	0.240
	Case	1.097	0.106	1.234	0.947		
IO R	Control	1.125	0.125	1.329	0.918	0.624	0.510
	Case	1.077	0.045	1.145	1.015		
IO L	Control	1.122	0.094	1.261	0.988	0.238	0.719
	Case	1.065	0.061	1.141	0.944		
EO R	Control	0.898	0.118	1.074	0.631	0.343	0.457
	Case	0.947	0.095	1.124	0.850		
EO L	Control	0.923	0.080	1.039	0.771	0.851	0.210
	Case	0.958	0.221	1.297	0.661		
EPQ-RA Neuroticism	Control	1.500	1.168	3	0	0.521	0.346
	Case	1.125	0.991	2	0		
EPQ-RA Psychoticism	Control	1.083	0.793	2	0	0.792	0.270
	Case	1.375	1.302	4	0		
EPQ-RA Extraversion	Control	3.750	2.050	6	0	0.624	0,267
	Case	4.250	1.669	6	2		
EPQ-RA Sincerity	Control	4.333	1.614	6	1	0.157	0.659
	Case	3.250	1.669	6	1		
TSK-11 Avoidance	Control	13.917	2.712	18	10	0.208	0.578
	Case	12.125	3.441	17	9		
TSK-11 Harm	Control	9.250	1.765	13	7	0.734	0.056
	Case	9.375	2.615	14	5		
BD I-II	Control	11.083	8.185	27	2	1.000	0.036
	Case	11.375	7.963	26	4		
STAI T/A	Control	26.750	5.496	35	17	0.521	0.141
	Case	26.000	5.127	34	21		
STAI S/A	Control	28.000	6.551	39	17	0.208	0.738
	Case	23.875	4.422	29	17		

Own elaboration (control group *n* = 12; case group *n* = 8); S_x_ (Standard Deviation); * Mann–Whitney test (Sig.: <0.05); BFS (Bristol foot score); FPI (foot posture index); BMI (body mass index); F (flexion); AbH (abductor hallucis); CSA (cross-sectional area); AbDM (abductor digiti minimi); QP (quadratus plantae); FDB (flexor digitorum brevis); IRD (interrecti distance); RA (rectus abdominis); R (right); L (left); TrA (transversus abdominis); IO (internal oblique); EO (external oblique); EPQ-RA (Eysenck Personality Questionnaire Revised-Abbreviated); TSK-11 (Tampa Kinesiophobia Scale); BDI-II (Beck Depression Inventory); STAI T/A (State-Trait Anxiety Inventory-Trait Anxiety); STAI S/A (State-Trait Anxiety Inventory-state anxiety). Bold values indicate statistical significance.

**Table 2:** Correlations between variables of the ultrasound morphology of the foot.

Foot Ultrasound Morphology Variables Correlations	r	Sig. (*n* = 20)	φ
BMI-SENSE F	0.568	0.009 **	0.753
BMI-IRD	−0.510	0.022 *	0.714
FPI-TrA R	0.600	0.005 **	0.774
FPI-IO L	0.514	0.020 *	0.716
BFS-HALLUX F	0.494	0.027 *	0.702
HALLUX F-TOES F	0.916	0.000 **	0.957
TOES F-CSA QP	0.451	0.046 *	0.671
SENSE F-CSA AbDM	−0.510	0.022 *	0.714
SENSE F-IRD	−0.459	0.042 *	0.677
THICKNESS AbH-CSA AbH	0.498	0.025 *	0.705
THICKNESS AbH-IO L	−0.540	0.014 *	0.734
CSA AbDM-TrA L	0.508	.0.022 *	0.712
CSA QP-RA R	−0.505	0.023 *	0.710
THICKNESS FDB-HEEL FAT	−0.498	0.026 *	0.705
CSA FDB-RA L	−0.525	0.017 *	0.724
HEEL FAT-TrA L	0.559	0.010 *	0.747
EO R-EO L	0.589	0.006 *	0.767

* Significant correlation at the 0.05 level (bilateral). ** Significant correlation at the 0.01 level (bilateral). BMI (body mass index); F (flexion); IRD (interrecti distance); FPI (foot posture index); IO (internal oblique); L (left); BFS (Bristol foot score); CSA (cross-sectional area); QP (quadratus plantae); AbDM (abductor digiti minimi); AbH (abductor hallucis); TrA (transversus abdominis); RA (rectus abdominis); FDB (flexor digitorum brevis); EO (external oblique); R (right); r (correlation coefficient).

## Data Availability

The datasets generated and/or analyzed during the current study are available from the corresponding author on reasonable request.
